# Retrospect of the Medical Sciences

**Published:** 1843-08-19

**Authors:** 


### DR. M'CORMAC ON PROLAPSUS ANI.

Reflecting on Dupuytren's procedure, it occurred to me that the same result might in a measure, at least while the child was at stool, be secured by careful manual traction. I immediately stated my views to the intelligent mother; she entered into them at once, and promised, if possible, to carry them into effect. According, when the child went next to stool, the skin exterior to the anus was drawn to one side by means of the fingers extended around. The little girl submitted to this with some reluctance, and complained that she could not evacuate her bowels. She was encouraged, however; a stool was obtained; from that day and date, now a month since, the bowel has not once descended. The stools, which previously were from two to four every day, have become much fewer, as well as of a more formed consistence and natural colour; while the child's health, spirits, and strength, are in other respects much ameliorated. There is now no prospect of the disease ever returning; the little girl requires comparatively little attendance; her mother, in fact, is only required to stand by, and in a short time, it is to be hoped, her onerous and anxious ministry will wholly cease.

As prolapsus ani is a very common affection coming frequently under the notice of every practitioner, and hitherto most difficult to remedy, this painless and bloodless mode of treatment, which, it is reasonable to suppose, would be equally efficient in other cases, in relieving the sufferings of helpless childhood, and lessening a mother's cares, seems deserving of attention.—*Dublin Journ.*

---

### EMPLOYMENT OF RESTRAINT IN THE TREATMENT OF INSANITY.

Some persons have gone so far as to suppose that all insane persons might be managed without restraint, a conclusion not warranted by reason, nor confirmed by experience. The system, indeed, of non-restraint has been so strongly advocated, and the public led to consider all restraint as cruel and unnecessary, and that a system of kind and watchful treatment is only required; that I cannot refrain from expressing my opinion, that, agreeable as this method would be to all humane and considerate persons who have the care of these unfortunate individuals, it will not *alone* avail. The minds of the insane must be operated upon by control, either in fact or in idea. The greater number may, and certainly ought to be, rendered tractable without bodily restraint; but we must attribute the orderly behaviour we are able to produce to a consciousness of being under restraint, which all patients feel when placed under the care of those whom they soon find they must obey, by being obliged, from the first, to take their meals and their exercise, to go to bed, and to get up at the appointed hours; and, but for this fortunate circumstance, we should have great difficulty in keeping an otherwise ungovernable patient in a state of quiet subjection.

Those patients who consider themselves sane and unjustly confined, as many do, use all the cunning they possess to elude our vigilance. The kindest treatment is no compensation to them for the loss of their liberty; and unless they are restrained, or taught to restrain themselves, would assuredly not remain in confinement. It frequently happens that the very threatening a patient with any particular restraint or discipline supersedes the necessity of its enforcement, and it is humane and prudent that this should first be tried; but, if positive restraint is required, it is for the ultimate benefit of the patient that the waistcoat should be used in preference to such half measures as I have seen recommended—viz., that "two persons should hold a patient in his paroxysm," which is neither more nor less than inviting him to struggle for the mastery. I have witnessed the inutility and mischief of this method, by having had patients brought to me covered with bruises, in consequence of the struggles that had daily occurred between them and their servants, all which would have been prevented by the more merciful restraint of the waistcoat, which is a most salutary remedy, as it at once puts the body into a comparative state of repose, and, by subduing the spirit, forces the mind to reflection, and leads to tranquillity, while the other creates contention and increases the excitement.—*Provincial Medical Journal.* June 3, 1843. *From Dr. F. Willis'. Treatise on Mental Derangement.*

### RETROSPECT OF THE MEDICAL SCIENCES.

### A FEW OBSERVATIONS ON ENCYSTED HYDROCELE.

BY ROBERT LISTON, F.R.S.,

Surgeon to the North London Hospital.

The author commenced by referring to the excellent manner in which the subject had been treated of by former writers, both ancient and modern, and stating as an apology for introducing it, that he had recently made an observation which induced him to believe that some collections in the scrotum are more intimately connected with the testicle, or its seminiferous tubes, than has been generally supposed.

When swellings of this kind have attained a large size it is difficult, if not impossible, to distinguish between the encysted and the common hydrocele; so that it is only when we have the opportunity of examining them at an early stage of their formation that we can ascertain their nature correctly. He adverted to the description given by several authors of eminence, of cysts formed in immediate proximity to the testis or epididymis, and pointed out some of the principal characters by which encysted hydrocele is distinguished from collections in the tunica vaginalis. The peculiarity of the former is, that the fluid contained in it is clear and limpid, and exhibits no trace of albumen; and another is, that in the operations for radical cure, its walls are not so prone to take on inflammatory action as those in common hydrocele. One object of the author's communication was to give a rational explanation of this circumstance.

About nine or ten months since, he was consulted by a gentleman beyond the middle period of life on account of tumour of the scrotum. There was plainly fluid on both sides. The largest cyst was punctured, and gave exit to some eight or nine ounces of this fluid, resembling distilled water, with a little soap diffused through it. The other side was punctured a few months afterwards, and five or six ounces of ordinary serum discharged. A short time since, the patient returned to have the first cyst again emptied. The fluid had the same appearance as before. It contained scarcely a trace of albumen. On the second day after it had been drawn off a small quantity was examined with the microscope, when it was found quite full of spermatozoa. It contained, also, some of the primitive cells, in which the spermatozoa are developed, and mucous globules. Had the fluid been examined sooner, probably the animalcules might have been found in motion.

The preceding observation was lately confirmed by the examination of the fluid withdrawn from a small cyst in the scrotum of a man aged fifty-three, who had applied to be treated for a bad stricture. It was nearly transparent and colourless, and was found to contain numerous spermatozoa, some of which continued to move actively for a considerable time after the fluid had been drawn from the cyst.

The author referred next to several preparations of cysts connected with the body of the testis and epididymis, which he had lately attentively examined with the view of ascertaining, if possible, the nature of the connection between these collections of fluid, and the glandular structure of the testis. He concluded by proposing the three following questions for invastigation.

First. Does the limpid fluid drawn from cysts of the scrotum, or inguinal region, uniformly, or often, contain spermatozoa?

Second. What connection subsists between the seminiferous tubes and these cysts?

Third. Whether dilatation of parts of the epididymis or vas deferens, from obstruction or otherwise, may not, in some instances, give rise to these collections?

If it be established that these cavities are always lined with mucous membrane, we should have an easy solution of the difficulty regarding a radical cure not following injection, as in the serous cyst.

Mr. Blizard Curling said, that the fact of spermatozoa being found in the fluid of encysted hydrocele was interesting and original. He himself had seen them in great numbers in this fluid. He was disposed, however, to question the explanation of the circumstance as given by the author. He doubted much if these cysts were formed by one of the seminiferous tubes. He had examined these cysts, and found them to partake rather of the serous than the mucous character. He should attribute the presence of the spermatozoa in the cysts to the accidental rupture of a seminiferous tube into them.

Mr. Cæsar Hawkins thought that the observation of Mr. Liston would require confirmation. Generally these cysts were free, and there was a distinct membrane between them and the seminiferous tubes. The same kind of cysts found in other situations of the body would often resist the ordinary means of cure by injection. They were constantly changing their character. They would at first secrete a thin watery fluid, and afterwards their coats would become fibrous, and the fluid secreted would be albuminous. He related the case of a woman in St. George's Hospital, who had a large cyst extending from Poupart's ligament some distance into the abdomen. It was moveable. Setons had been frequently passed through it without producing any beneficial effect. It was accordingly determined to extirpate it. It was traced, on making an incision over it, to the femoral ring, and found to be a hernial sac, blocked up by a piece of omentum. It no longer secreted serum, but merely a watery fluid. This explained its resistance to the ordinary means of cure.—*London Lancet.*

---

### EFFECTS OF MENSTRUATION ON THE SECRETION OF MILK.

At the Academy of Medicine, Paris, May 31, 1843, M. Raciborski read a memoir on this subject. His conclusions were drawn from seven cases of women who continued to menstruate during the whole or greater part of the period of suckling; they are—

1. That, contrary to the generally received opinion, the milk of nurses who menstruate during the period of suckling does not differ in any appreciable manner from that of nurses who do not menstruate.

2. The only difference, worth noting, is that it contains less cream—a fact on which the blueish colour of the milk from some women depends.

2. That the inconveniences of allowing a woman to nurse during menstruation has been greatly exaggerated, and that a nurse should never be rejected on that account alone.—*Prov. Med. Journ.*

### ANTIDOTE FOR THE BICHLORIDE OF MERCURY.

M. Orfila, having lately performed a series of experiments, with a view to determine the value of proto-sulphuret of iron as an antidote for corrosive sublimate, has published, in the "Journal de Chimie Medicale," the results at which he has arrived.

1. That the proto-sulphuret of iron completely destroys the poisonous properties of corrosive sublimate, if administered in sufficient quantity *immediately after* the ingestion of the poison.

2. That, like the other most approved antidotes for this poison, it is inefficacious, unless given within about ten or fifteen minutes, by which time the deleterious action of the poison is generally sufficiently powerful to cause death.

3. That, although the sulphuret decomposes the corrosive sublimate more powerfully and completely than *albumen*, and, therefore, ought to be preferred in all cases where it can be administered *immediately*, or in *a very short time*, after the poison; yet it will almost always be found that *albumen* may be more advantageously resorted to than the sulphuret of iron, in consequence of the latter being kept only by the pharmaceutists, and much time being, therefore, lost before it can be administered, while white of egg mixed with water, which is everywhere to be obtained, may be given without delay.—*Ibid.*

---

### SIR HENRY MARSH ON REGURGITATION.

This affection is, in many instances, one of the innumerable varieties of hysteria; this form of the disease I have met with more frequently than any other. It is oftentimes accompanied with tenderness, on pressure on either side of the spine; sometimes it exists alone, but more frequently it co-exists with other hysterical and nervous symptoms. In many of these cases there is a sense of distention and oppression, antecedent to the regurgitation of the food, which is completely relieved by this act. It is usually the solid part of the food which is rejected, and only as much as is necessary to remove the sense of oppression. This form of the disease affects young females principally; it is an affection of the nerves of the stomach, and by no means a dangerous disease, though sometimes obstinately resistant of treatment. It is often preceded by other disturbances of the nervous system, such as intense headaches, and various forms of neuralgia; violent and long continued palpitations; spasmodic cough and dyspnœa; convulsive affections of the muscular system; nervous vomiting and diarrhœa; and seems to take their place and supplant them. In these cases of purely hysterical and neuralgic regurgitation, the treatment I have found most frequently beneficial, consists of small blisters applied simultaneously to the pit of the stomach and to the spine; this, in some few instances, has been at once and permanently effectual; more generally, however, its salutary effects are only temporary; small detractions of blood by cupping in both situations have in some instances been equally successful. Benefit has been derived from small, and often-repeated doses of hydrocyanic acid, belladonna, morphine, stramonium, and other narcotics. Tonics, such as iron, bark, and bismuth, have also occasionally been found useful; but no remedy has been so often successful, in this form of the disease, as a total change of habits, change of air and scene, and travelling. In one case electricity was completely effectual in curing the complaint; it failed in others. In another instance, a sudden and powerful mental emotion totally and permanently removed the disease.

I have generally found it useful to advise for the patient the recumbent position for an hour or more after each meal; to eat slowly, and to masticate well the food; to eat less than the appetite demands; and to be abstinent in the proportion of fluids, so as to avoid distention of the stomach.

In two instances of pregnancy, I have observed that the sympathetic disturbance of the stomach, which so frequently characterizes that state, assumed this particular form; and much of the food taken in the early part of the day has been expelled, not by vomiting, but by regurgitation without nausea.

In not a few cases of tubercular disease of the lungs, this peculiar affection of the stomach has co-existed.

The regurgitation of the food without nausea is sometimes one of the many symptoms which mark the existence of a serious affection of the stomach. In such cases the food regurgitated is already partially and imperfectly digested; imparts to the mouth an acrid, bitter, or intensely acid taste, and is accompanied with the constitutional signs of materially impaired assimilation; such as loss of flesh and strength, pallid countenance, depressed spirits, mental misery; diminishing muscular and intellectual capability, loaded tongue, loss of appetite, or craving and gnawing at the epigastrium, irregular bowels and morbid excretions, and disturbed and unrefreshing sleep.

This form of the disease is generally traceable to long continued mental anxieties; too over thoughtful, studious, sedentary, and solitary habits, to the swallowing of food hastily without sufficient mastication and insalivation; to the utter neglect of the two most excellent promoters of healthy digestion—cheerful society, and full, free, enjoyable muscular exercise.

In some cases it may be traced to excesses in sexual indulgences, and abuses of the sexual propensity. Finally, every violation of the laws of nature, which tends to break down the vital energies, is capable of giving rise to this affection of the stomach.

Of all the predisposing causes, that which is most frequently operative is the existence in the constitution of the strumous diathesis.

One of the most important duties of a physician is to trace morbid actions to their source, as thus, in many instances, the method of cure is at once indicated; and in no form of disease is this truth more strikingly illustrated than in that now under consideration.—*Dub. Journ. of Med. Sci.*

---

### MR. HAMERTON ON ACUTE GLANDERS IN THE HUMAN SUBJECT.

Fever of six or eight days' duration precedes the eruptive stage of this disease. The patient suffers from severe rigors, headache, irritability of stomach, and thirst, lassitude, oppression of strength, and quick pulse. He also particularly complains of severe pain in his limbs and back, and also of stiffness, and heat of his joints. The disease at this early period might be confounded with acute rheumatism, and that it has so been mistaken and mixed up with it, cases are not wanting to prove the truth of the assertion. In the fever ushering in the acute form of glanders and farcy, the condition of the skin is remarkable: it has a dry, harsh, burning hot feel; in no instance, that I have observed, has there been any appearance of moisture in this first or premonitory stage. The contrary is usually observed in the fever of acute rheumatism, since perspiration is one of the characteristics of both its early and advanced stages. The appearance of the countenance of the patient in acute glanders is also very remarkable, and indicative of serious organic change, his face is pale, anxious and depressed, his features are sharp and contracted, there is a peculiar uneasiness and despondency in his manner; as the fever advances, it lapses into one of a low typhoid type; the pulse is small, quick, (in one case 160 in the minute,) and undulating; the tongue is dry, brown, and tremulous; diarrhœa and involuntary evacuations mark this advanced stage; there is also tremor of the limbs and subsultus, besides raving, low delirium, stupor, and coma. At this second or typhoid stage of the disease, the local characters of glanders and farcy present themselves to our notice, and it appears, that the more profound the prostration, so in proportion the different crops and forms of eruption advance to maturity, not simultaneously, but at each day's visit a fresh crop is presented to our view. The arrangement of the different forms of eruption I shall next proceed to state.

First. We have inflamed surfaces, varying in extent from two inches to the entire length of a limb, engaging the deeper structures, œdematous, and having an erysipelatous appearance, presenting elevations in parts, and yielding a boggy, inelastic feel on pressure, terminating in complete sphacelus, and giving out an insupportable fœtor.

Secondly. Superficial and subcutaneous pustules, circular or oval in shape, appearing in great numbers and in succession, becoming fully matured in a few hours, and containing a serous fluid, beneath a white crust of cuticle, without either surrounding redness or swelling.

Thirdly. Small erythematous patches of redness situated over joints, ending in sphacelus; the point of mortification commencing in the centre of a hardened base: diverging from it in all directions, purplish rings are to be observed.

Fourthly. As an advanced symptom and appearing towards the close of the disease, a discharge of yellow, tenacious mucus, changing into one of a dark, sanious character, from one or both nostrils: and,

Lastly. Inflammation of the lymphatics and absorbents is observed to be extensively in the vicinity of, and leading from, the gangrenous pustules.

That glanders and farcy are communicated by contagion from the horse, mule, or ass, to man, I have no doubt, and consider the above cases fully warrant such a conclusion; we find the subjects of the above cases constantly about the diseased animals, and that for some time their business was to clean harness, and feed the animals day and night; also in two instances an attempt at cure was tried in vain, and by the very persons who afterwards fell victims to this baneful disease; and although it does not appear that any abrasion or sore upon the hand was observed, still, when the habits of the peasantry are considered, their utter carelessness and want of cleanliness, after handling these animals, a portion of the glandered matter might readily find ingress into the system through the nasal mucus membrane; also, very few of the peasantry are free from fissures in the palms of the hands and fingers, by reason of their hard work, and this also might be another mode of the introduction of the virus of glanders into the system. It will appear, also, from the history of the above cases, that all indiscriminately do not fall victims to this disease, as in the case of Keegan, whose business it was merely to drive, and make up the diseased horse; his system was infected with the disease, and rapidly sunk under it, and the two persons who were constantly about the horse, applying remedies for its cure, escaped it. Can this disease be communicated from one individual to another by infection? In the detail of the cases, I have been particular in describing the state of the dwelling in which I found my patients residing—close, ill-ventilated cabins, without light or free circulation of air, with a number of persons residing in them, and the patients themselves continuing in the same clothes throughout the disease; in fact, every circumstance was here present to taint and vitiate the atmosphere around the patient; and still no one, of either friend, relative, or nursetender, sickened of the disease; and when the same state and condition of cabins is considered, which facilitates the spread of disease, both of an infectious and contagious origin, as small-pox, &c., and yet in no instance have any one of the numerous friends and relatives about the patients, labouring under acute glanders, taken the disease. I think it may be so far safely conceded from the above facts, that glanders is not an infectious disease, or likely to be communicated from one person labouring under the disease to another. This opinion is also further confirmed by what occurs in the history of the disease in the horse tribe. The majority of the most celebrated veterinary surgeons of the past and present period have declared it as their opinion, that glanders and farcy are diseases imparted by contagion alone, and that early separation of the diseased animals from the healthy, proper care, and requisite attention to cleanliness, will prevent the spread of the disease in them. Glanders and farcy have been divided into the acute and chronic form. The horses, from which my patients received the contagion of the disease, laboured under the latter form, and it will appear from the history of the cases, that all were some time in constant attendance upon the diseased animals, before becoming tainted with the poison of glanders, a circumstance admitting of an explanation, inasmuch as diseases imparted by contagion may be weeks, even months, dormant in the system, before unfolding themselves to our notice. The disease appears to pass through the human system unaltered, or in any way modified in its characters, in fact retaining its baneful and destructive properties, as is proved by the experiment upon the ass; on the second day it became ill; on the fifth it died. Lastly, in the absence of any therapeutic means of curing, or even alleviating, this so formidable and fatal disease, one in which the records of medicine have not furnished a single instance of recovery, and one also in which the physician is unable to afford an interval of ease, or assuage the most frightful agony that human nature is exposed to; with all these facts before us, and in the humiliating reflection of being merely silent lookers on, and all the resources of our art in this disease futile and useless, we should turn our thoughts to preventive measures, and impress upon the attention of the local authorities in our respective districts, the dangerous and fatal consequences likely to ensue, by allowing glandered horses to exist, or be suffered to go at large.—*Ibid.*

---

### DR. SUTHERLAND ON THE BLOOD IN INSANITY.

There remains for us to consider the quality of the blood in mental alienation: so intimate is the connection between the capillaries and the ultimate filaments of the nerves, both at the periphery and at the centre, that what affects the one is likely ere long to affect the other. Not only do we perceive this in the blush of shame and the paleness of anger, and in the effect which the passions have upon the action of the heart, but we see also that injury to the nerves produces stagnation in the capillary tubes, thereby of course greatly interfering with nutrition and secretion. The division of the par vagum, we learn from the experiments of Dr. Reid, is followed by disturbances of the latter, arising from paralysis of the nerves of the stomach, and not, as stated by Dr. W. Philip, from want of secretion of the gastric joice, such secretion being in no wise impeded. It is not material whether the alteration in the quality of the blood is the cause or consequence of the disease. The alteration of the blood is the cause of the disease when it is deprived of the qualities necessary for supplying proper nourishment to the brain, and also when, owing to the skin, the uterus, the liver, or the kidney, not performing their proper functions, it is not properly elaborated. On the other hand the alteration is the consequence of the disease; for though nutrition and secretion are not dependent upon the nerves, yet they are materially influenced by them; and in this and other disorders of the nervous system the circulating fluid must undoubtedly suffer.

Familiar instances of the influence of poisoned blood upon the brain are to be found in the effects of alcohol and opium. No less striking are the consequences which follow when the elements of the blood are not repaired by proper nutrition. "In the progress of starvation," says Liebig, "it is not only the fat which disappears, but also, by degrees, all such solids as are capable of being dissolved. In the wasted bodies of those who have suffered starvation, the muscles are shrunk and unnaturally soft, and have lost their contractility; all those parts of the body which were capable of entering into the state of motion have served to protect the remainder of the frame from the destructive influence of the atmosphere. Towards the end, the particles of the brain begin to undergo the process of oxidation, and delirium, mania, and death, close the scene; that is, to say, all resistance to the oxidizing power of the atmospheric oxygen ceases, and the chemical process of eremacausis, or decay, commences, in which every part of the body, the bones excepted, enters into combination with oxygen." Chemical analysis has not aided us at the present day in detecting any characteristic peculiarity in the brains of the insane. The brain of man is chiefly composed of albumen, a large proportion of water, and a peculiar fatty matter. The latter presents us with results of most interest; it was in this that phosphorous was detected by M. Vauquelin. M. Couerbe, following up these experiments, thought he could appreciate distinct variations in the quantity of phosphorus in the brain of the sane and insane. In the former he asserts that it is present to the amount of from 2 to 2½ per cent.; in the brains of violent maniacs to that of 3, 4, and 4½ per cent.; while in those of idiots he says that it is deficient in quantity, being not more than from 1 to 1½ per cent. The value of these experiments has been rigorously tested by M. Frémy, in a very interesting paper published in the 3d series of the Annales de Chimie et de Physique. "All my efforts," says he, "have been employed in studying the fatty matter which can be obtained by digesting the brain in alcohol or ether. It was after evaporation with ether that M. Couerbe discovered cholesterine, the white substance containing phosphorus, which he calls cérébrote, and moreover three fatty substances which he considers neutral, and which he calls céphalote, stearoconote, and éléencephole; (see Annales de Chimie, tom. lvi. p. 164.) The results which I have obtained differ in *every* point from those just quoted, for I have not seen in the substances of M. Couerbe anything but an acid fatty agglomeration in combination with a saponaceous compound. Setting aside the fatty substances which are found common to other animal matter, we find that the brain is characterized by the presence of cholesterine, and by two peculiar fatty acids. This composition is much more simple than M. Couerbe has been inclined to admit. M. Frémy goes on to state that, in fresh brains, the oleophosphoric acid is found, but that it soon becomes decomposed, and in a brain which has not been examined for some days, you find phosphoric acid in a free state, and oleine. M. Vauquelin found the same alteration in ramollissement of the brain, which he considers to be exactly analogous to what takes place in putrefaction. It is interesting in connection with this subject to know that M. Chevreul has found in the blood the fatty matter of the brain, and M. Boudet cholesterine. M. Vauquelin has, also, in some cases, found the peculiar fatty matter of the brain in the liver, which is certainly a discovery of much importance: it may, perhaps, throw some light upon the subject of sympathy between the brain and the liver. We must rest contented for the present with the theory of sympathy to explain the mutual action and reaction of the central organs of the nervous system with each other, and with the nerves themselves. Esquirol lays much stress upon this subject, and draws our attention to the connection existing between the ganglia of organic life and the brain, as leading us to trace the source of the disease to the ganglia. The central parts of the nervous system do, as we know, sympathize with the functional and organic disturbance of all organs of the body. The opinion, therefore, that insanity is sometimes a sympathetic disease, is not so very improbable as some have supposed; but, whether sympathetic or idiopathic, it becomes a matter of great importance in practice to consider the diseases with which insanity may be complicated, and to relieve the symptoms not only which take their origin from the disease of the brain, but those also which are due to the disease existing at the same time in any other organ, as the mental disease will undoubtedly be much aggravated if attention is not paid to the latter. As there is no disease in the body with which insanity may not become involved, so there is no medicine in the Pharmacopœia which you may not have at some time or other to prescribe in treating mental affections. The search after specifics for the cure of madness does, therefore, appear to me to be as vain as the labours of the alchymist to discover the philosopher's stone. Although there is no disease with which insanity may not be complicated, yet there are some more commonly met with, in conjunction with it, than others; *e. g.* scrofula, phthisis, inflammation of the lungs, disease of the heart, congestion of the liver, diseased kidneys, inflammation of the mucous lining membrane or the intestines, diarrhœa, erysipelas, epilepsy, apoplexy. Sometimes an attack of diarrhœa, of phthisis, or of fever, will put a stop to the attack of insanity; sometimes the attack will be merely suspended. The approach of the catamenia is always a critical period with our female patients; the disease is then often aggravated, and paroxysms of furor occur; the return of the monthly period sometimes gives an intermittent character to the disorder, and the hopes of the friends and of the physician are alternately raised and depressed. It is not uncommon to meet with insanity manifesting itself in one member of a family, while nervous disorders akin to it develope themselves in the rest. This takes place also, occasionally, with respect to other diseases, which are not of a nervous type, *e. g.* scrofula and phthisis. A patient under my care, who died of general paralysis, was the only one of ten children who did not die of phthisis.—*Lond. Med. Gaz.*

---

HEMIPLEGIA FROM TYING THE COMMON CAROTID ARTERY.

M. Sedillot applied a ligature to the common carotid to arrest hæmorrhage, in a man who was wounded behind the right branch of the lower jaw. Complete hemiplegia of the left side of the body, and of the right side of the face, followed, and the patient lost his intelligence so far that he could scarcely comprehend questions put to him. He died nine days after the application of the ligature, and the post-mortem examination showed that the hemiplegic symptoms had resulted from the right side of the brain, having been deprived of its due proportion of arterial blood.—*Gazette Médicale;* and *American Journal of the Medical Sciences.*

---

[The subjoined notice of Dr. Wilson's work on Spasm, Langour, Palsy, and other disorders termed Nervous, is from the London Lancet.]

The little volume of Dr. Wilson contains a great deal that is philosophical and much that is interesting; but we cannot admit of that exclusiveness of action in the production of phenomena which the author is inclined to attribute to the blood alone; and when he states that "We often hear of spasm in structure where no muscle has been demonstrated by the anatomist," we may state that the anatomist himself has yet much to do; and if we allow of muscularity in some structures, on the authority of their functions, we may ascribe spasm to their existence in others, excited, as it may be, by the influence of blood or nerve.

Dr. Wilson has found, in his own experience, that those fevers which are ushered in by spasm become eruptive in their progress, and instances cases of spotted epidemic fever, and chicken-pox, in corroboration of his assertion. This manifests, by anticipation, the strong tinge of humoral pathology with which these pages are imbued. The author lays no claim to novelty in the statement just quoted, as it is a well-recognized fact; for Cullen observes that small-pox is frequently ushered in by an epileptic fit, and Dr. Gregory laid very great stress upon this curious circumstance in his lectures on the exanthemata.

We require to be reminded of such important pathological truths; for the rarity of the small-pox since the introduction of the vaccine has allowed, perhaps, but few persons to become practically acquainted with them; and the experience of others glides away from our memories, unless the impress is renewed by occasional repetition.

The influence of mercurial vapour in producing convulsions and paralysis admits of more general observation, and is no where better exemplified than in the sad condition of the workmen who are employed, for instance, in the gilding of the cupolas of churches, of which we have seen many and lamentable instances. The gold is laid on with mercury, and the latter volatilised by heat, and many a sturdy fellow is doomed, in the prime of life, to drag on a miserable existence for the remainder of his days, shaking with palsy, unable to convey his hand to his mouth, after the inhalation of this poisonous vapour.

These are some of the instances which are adduced of spasmodic action and convulsion, caused by the influence of blood deteriorated by the diffusion of poisons through its mass.

"Spasm, in all cases, excepting those of direct local injury, implies a further effect of disorder in the system than is expressed by the irregular contractions of the affected muscle." * * * "In shaking palsy induced by mercurial poison the spasm is of this pervading kind. It is but one symptom among many of the constitutional disease, and is associated with evidence of disordered action in every part of the system."

Cramp, which affords the best illustration of spasm in the double voluntary muscles, is not considered by Dr. Wilson as ever deserving the name of an idiopathic affection,—that is to say, occurring without constitutional disturbance. After stating that spasm is not necessarily connected with disease in nervous structures, nor with abscesses in the brain, tumours, or ossification of the membrane, the author assumes that "it is from what offends the blood in that fibre that spasm of the voluntary muscles is for the most part induced? There are no cramps," he adds, "so severe as those of which poison mixed with the blood is the direct cause." This affords the author an opportunity of reviving the Hunterian doctrine of the life of the blood, which he does in very energetic language:—

"Can we refuse the inference that always in the muscles, when thus disturbed, there is a prejudice of the blood by composition or in function, and by the blood's function how much is implied—how much more than the little we know of it by its material composition or in its mechanical relations? This has not been enough considered. The blood not only lives, but its life predominates over that of all else in the system. It lives in the mass as in the particles, as an organ, that is, with a combined function; as an organ, therefore, universal in the body; for the blood in the living body is everywhere, and everywhere at once. As the air surrounds us from without, so does the blood pervade us, like an atmosphere, from within, receiving and transmitting influences through its continuous mass, which are acknowledged in all parts of the system. Most of what is effected by the blood is thus accomplished,—not by the direct admixture of material principles with its current, and the subsequent transport of such principles to the several organs,—not by chemical agencies thus conveyed,—but by action inherent in the blood itself, rapid and universal as electricity, occasionally developed by the mere contact of certain forms of matter, or by agencies that are immaterial."

Again,—"It is not so much from what is added of *material* to its composition, as by what it receives of impulse to its action, that effects follow from the blood to the system. Cramp is among those effects, which are most frequent in those structures where, as in the muscle, the blood is most abundant. Self-moving, self-producing, maintaining its own fluidity, arresting its own current by self-coagulation, the blood, in its wide range of capacity, is affected directly and at once by the countless agents of vital impression. Hence, from these varying states of blood in the flesh, proceed the various symptoms of disturbed action that are usually termed 'nervous,' in the voluntary muscles."

Although we assent to the principle, as a general proposition, that the blood possesses properties which place it in a very different light from fluids passing mechanically through tubes, but which are endowed with no special qualities, still we are not prepared to attribute so much to the blood alone, nor to endow it with such independent properties. We must remember that that fluid is propelled through living organized tubes, which retain their vitality after the blood that escapes from them loses it, and that the blood becomes *defunct* as soon as it is effused,—is decomposed, is an *extraneous body*, is *a heap of refuse*, and requires the operation of *vital scavengers* to remove it. It is positively certain that noxious matters diffused through the blood do excite spasmodic action in muscles, and we cannot admit the occurrence of spasm where no contractile fibre exists. Still, we have seen many cases of idiopathic spasm where there can have been no question respecting the existence of deteriorated circulating fluid. In their younger days many persons never dance without suffering subseqnently from cramp in the calves, and still have the misfortune to be severely pinched in drawing off long boots, being compelled to halt with one leg in the boot-jack. Indeed, some individuals can produce cramp, at any time, in the great toe, by bending it down upon the sole of the foot. In all such cases we would ascribe a due share of influence to the blood, but that only of a mechanical kind. To the nerve which throws the muscle into contraction we must look for the *primum mobile*, and we think that Dr. Wilson has philosophically suggested the cause of the pain when he says that "it may possibly depend on the contraction in opposite directions of unequal segments of the affected muscle." We certainly believe in the existence of idiopathic cramp where the blood is in its natural state, but consider that it is excited by a deteriorated condition of that fluid, and that, as the author justly observes, "There are no cramps so severe as those of which poison mixed with the blood is the direct cause." No spasms were more terrific than those that were witnessed in some cases of the cholera, a disease arising, as we fully believe, from a specific poison diffused through the blood, producing its malignant effects on the nerves and muscles. We once witnessed the cure of a paralysed limb by the violent universal spasms which occurred in a case of cholera. An old lady had almost lost the use of her left arm for years, and reported that she had suffered a slight paralytic stroke. She was attacked with spasmodic cholera in its most painful form, the cramps in the arms and legs being most severe, but she finally recovered, and completely regained the use of the paralysed arm.

Muscle, nerve, and blood, form an indissoluble trinity, and healthy action depends upon the due proportion of labour allotted to each. It is impossible for one to be deranged without affecting the others. Action and reaction may occur between them, producing derangement, or, as we have seen, restoring function. The paralytic limb loses part of its animal heat, its muscles become flabby, and its circulation less active, and the blood which flows through it may feel this impress, but the palsy may have been induced by mechanical means, the removal of which is followed by a restoration to its normal condition. The nerve shall regain its irritability, the muscle its tone, and the blood its momentum. In other cases the blood, poisoned by extraneous matter, as by strychnine, shall become the prime offender, and acting upon the nerve and muscle, excite them to extraordinary action. Still they are but meshes of the same web. Tread upon a fibre and the whole vibrates.

Dr. Wilson wishes to rescue *tetanus* from the class of the *neuroses*:—

"By thus considering tetanus in its wide and constant relations with the blood, we cannot fail to know it in its true character of a great constitutional disorder. Because it begins and ends with spasm of the voluntary muscles, tetanus has hitherto been classed with affections of the 'nervous system,' an undue limitation of the disease, for which there is no sufficient warrant by the symptoms, or in its general pathology. Like fever, it pervades the entire system, and is special in the flesh, being common in the blood. A fever in truth it is, spasmodic and remittent in its character. As a fever, it proceeds from local injury or constitutional disturbance, requiring for its full development a certain period of previous incubation. * * * * In familiar phrase, tetanus may be termed the *muscular fever* of pathology."

We doubt whether this will meet with general assent as regards tetanus in its traumatic form, which the author does not exclude in his general view of the disease. It is here that we obtain the fullest view of the author's opinions. Especially in this part of the volume is it that the challenge is given,—Blood *versus* Nerve:—

"By no irritation, by no torture, however ingeniously devised, of any nerve, or of any number of nerves, in the body, would it be possible to establish a complete tetanus in the muscular structure under a given period of time, varying from forty-eight hours to three weeks."

What all the tortures of the rack and screw cannot accomplish under a certain number of hours, (forty-eight being the minimum,) is produced in as many seconds through the medium of the blood:—

"We have seen, on the other hand, that in a few seconds, a tetanic spasm, universal and fatal, of the entire range of the voluntary muscles, may be induced by the introduction of a few drops of poison to the blood."

This is sanctioned by all the experiments which we have seen where poison has been introduced into the veins of animals. Still, it is upon the nerves that the poisoned blood produces that influence which causes cramp and spasm, these also being produced, if not so readily, still more quickly than the author admits, by simple lesion of a nerve. Dr. Gregory used to relate the case of a waiter whose thumb was lacerated by a piece of an earthen dish coming out as he lifted it from the table; spasms, convulsions, locked-jaw, ensued, and he died on the same evening.

We must not indulge longer in discussions which seldom lead to conviction, and only lament, with Fontenelle, that we have not better eyes to help out our philosophy, seeing through all our magnifying glasses but very darkly as yet. Let us observe more closely, studying the facts which present themselves —and this volume abounds in facts of very great interest.

The author directs attention to renal disease and its consequences, and affords proofs of the neglect of due investigation and want of proper discrimination in treating those cases. It is very satisfactory to quote such passages as the following:—

"Whoever has seen a patient comatose from retention of urine is aware of this dependance of the brain for the proper discharge of its function upon the kidney; yet few care to understand how rapidly, entirely, and fatally the gland may influence the nerves in their central assemblage, which is the brain."

It is surprising that the experiments of Darwin, when prosecuting his inquiries under the impression of discovering a direct communication between the stomach and the bladder, should not have directed the attention of pathologists to this important point. The ureters of dogs were tied with ligatures; the animals soon became convulsed and died, and, upon opening the head, serum was found effused upon the brain.

The volume contains many things that are deserving of perusal and investigation; but we must conclude, at present, with this important quotation, our limits not allowing us to treat upon many practical points, such as the treatment of cholera, to which Dr. Wilson directs attention:—

"My inference from these cases of sudden death from renal disease, coincident with integrity of the cerebral structure, is, that in the treatment of epilepsy, anasarca, and hydrothorax, of apoplexy, and disorders of the pulse and breath, the structure and function of the kidneys should be taken well into account. Moreover, that in all dissections of persons who have died suddenly, the kidney should be carefully examined; and this, whether the heart, brain, lungs, and large blood-vessels be sound or not; for disease of the kidney is often the cause of organic disease in the vital structures, influencing their nutrition through the common material of the blood, upon the elaboration of which the kidney is unceasingly employed."

---

### INFLUENCE OF EXTERNAL AGENCIES ON DISEASE.

The influence of climate, diet, and other physical circumstances in modifying the organisation of man, has long been made a subject of observation by physiologists.

These, however, affect not only the *healthy* functions of the body, but also its *morbid actions*,—both the symptoms of disease and the operation of remedies. Manifold remarks have especially been made relative to their share in producing diseases that are endemic to *particular* countries. Much less attention, however, has been given to a point of still greater moment, namely, the influence of climate, diet, and various physical agencies in modifying diseases which are common to *all* countries. The importance of this inquiry is sufficiently evident from the serious errors into which pathologists have fallen from not keeping it in view.

Take the example of that most frequent and formidable disease, *continued fever*. This malady, as it existed in London a century or more ago, was of an exceedingly low type, and frequently attended with petechiæ, vibices, passive hæmorrhages, and other indications of what has been called a dissolved state of the blood, whence it was commonly termed "putrid fever," and generally believed to consist, peculiarly and essentially, in "a morbid state of the fluids." In the present day such a case is hardly ever seen in London. Even the lighter forms of typhus are extremely rare, and the prevailing type of continued fever is *synochus*. Yet no reasonable doubt can be entertained that the putrid fever of Sydenham's days was essentially the same disease with the synochus that is now prevalent.

Again; the enlargement and ulceration of Peyer's and Brunner's glands at present so uniformly appear in the bodies of those who have died of continued fever, in *France*, that many able pathologists of that country regard these as the essential features of the disease; but those pathologists would find that in *London* they might dissect hundreds of fever cases without encountering a single instance of the lesion referred to; they would find, moreover, local complications of fever to be more frequent within the cranium than in the abdomen.

Once more: the type of continued fever in *Dublin* and *Glasgow* is at present much more typhoid than in *London*, and more so in Edinburgh now than it was in the same city *some years since*.

It hence becomes obvious that pathological conclusions, arrived at from the contemplation of fever in any *one* period or locality, are nearly certain to be premature and erroneous. The influence of climate, diet, habits of life, and endemic and epidemic causes, require to be taken into account, and we must not presume that we have arrived at a satisfactory theory of fever until we have found one resting on facts that have been invariably observed on correct principles.

This truth is strikingly exemplified in the late progress of pathological opinion with respect to continued fever. The hypotheses of Clutterbuck and Broussais, referring the disease to inflammation of the brain, or the gastro-enteric mucous membrane, have taken unto themselves wings. The more recent *dothenentérite*, although still supported by high authorities, is evidently losing its hold, and judicious pathologists are becoming strongly inclined—reverting to the old view—to regard continued fever as the result of some cause acting principally, if not primarily, on *the blood*. Thus, after a century of partial observation, the theory of fever is likely to return to the point at which it set out. Happily, however, in this, as in other instances, observation has not been fruitless, though unsuccessful with reference to its immediate object; for, just as the labors of the alchemists developed the facts of chemistry, although they did not discover the philosopher's stone, so the observations of the morbid anatomists, notwithstanding that they have not unveiled the essential cause of fever, have made known a variety of local complications, on the knowledge of which the successful treatment of the disease in a great measure depends. But if the anatomical inquiries had been prosecuted in a more philosophical manner, we should have had the benefit of the same facts without the encumbrance of the false theories, which never could have been entertained had pathologists been careful to correct their own observations by those of others, made at different periods and places.

The same holds good with other diseases. Pathological states that are *essentially the same*, vary somewhat in *different times* and *places*, both in the phenomena which they present and the treatment that they require.

For instance: take dyspepsia as it occurs in London and in Paris. In the latter city, the disease is particularly liable to be accompanied by chronic gastritis, which is probably owing to the large consumption of ascescent wines and coffee, both of which have a great tendency to irritate the gastro-enteric mucous membrane. This has possessed the Broussaian school with the notion that there is no such thing as dyspepsia apart from chronic gastritis; and the adoption of this erroneous notion has done more harm in England than in France; for, in the latter country, chronic gastritis is frequently a prominent feature in the state that is called "dyspepsia," which is not the case in England, although it sometimes occurs there; and hence young gentlemen, fresh from Paris, often prescribe for their English patients leeches to the epigastrium, and abstinence from animal food, where a nutritious diet, tonics, and country air, would be much more to the purpose.

If physical causes occasion diversities in the type of *disease*, it may readily be conceived that they exert no less influence on the operation of *remedies*. It is usual to talk of "the fashion of medicine;" and, doubtless, our art, like other sublunary things, is swayed by caprice and the love of novelty; but a change of views with respect to the applicability of certain remedies may be traced to a deeper source, namely, *an actual change in the type of disease*, and a consequent variety in the relation of therapeutic agents to morbid states, which, although nominally, are not actually, the same. Thus when, for a series of years, diseases in general have maintained a sthenic character, blood-letting comes into vogue; but let a low type of disease become prevalent, and the lancet gets into disgrace, and experienced gentlemen shake their heads, and say that "men grow wiser as they grow older." In like manner, if an irritable state of the gastro-enteric mucous membrane happen to be frequent, purgatives fall into disrepute, but recover their reputation when the intestinal tube becomes less susceptible of impressions.

Thus, in occasionally drawing attention to the improvements which it is necessary to make in medical practice, as time rolls on and knowledge advances, we would urge on the community of medicine the importance of giving attention, amongst other things, to the influence of external agents on morbid actions and the operation of *remedies*. The attainment of a perfect knowledge on this subject is extremely difficult, and can be hoped for only in the most enlightened state of the profession throughout the world. Meanwhile, much may be accomplished by diligent comparisons of medical experience among different nations. Military and naval surgeons, in particular, have great opportunities of promoting the object in question, after witnessing the aspects of disease in the various climates that they visit, and the diversified circumstances which attend the periods of their observation.—*Ibid.*

---

### SPONTANEOUS CURE OF OVARIAN DROPSY.

The following case is recorded by M. Hay, at Altena, in Prussian Westphalia: A woman, aged forty-eight, who had previously been in perfect health, was the subject for some time of great uneasiness in the hypogastrium, when at length, on the right side of the abdomen, immediately above the ramus of the pubis, there appeared a large tumor, somewhat moveable, and unequally distending the abdominal parietes. The accompanying symptoms were pain in the thighs and the right leg; the lower extremities œdematous; dyspnœa, &c. The clear diagnosis furnished of ovarian dropsy had induced the practitioner to advise the operation of paracentesis, which was on the point of being performed, when a large serous discharge issued from the vagina, and lasted about four days, at the close of which time the tumor and all its concomitants disappeared. It shsuld be mentioned that this affection had no influence either in stopping or diminishing the menstrual discharge; only one ovary, therefore, appears to have been affected.—*Ibid, from Medicinische Zeitung.*

### NEW MODE OF ADMINISTERING MERCURY, AND THEORY OF ITS CHANGES IN THE SYSTEM.

M. Mialhe is considered to have established that all the preparations of mercury employed medicinally act by being changed in the animal economy into the bichloride, to the action of which preparation their therapeutic and toxicological effects are owing. This chemical change is produced by the alkaline chlorides present in the humors of the animal frame. The proportion of bichloride thus formed is partly in correspondence with the quantity of the alkaline chlorides present; but partly, also, dependent on the kind of mercurial preparation taken,—the deuto-salts being at once converted into corrosive sublimate, while the proto-salts pass first into the state of proto-chloride, and only attain their final composition and efficiency by a secondary change. Finally, the bichloride of mercury forms, in conjunction with the alkaline chlorides and the albuminous materials of the blood, a chemical combination susceptible of traversing the whole vascular system without appreciable alteration.

Acting upon these data, M. Mialhe, in order to place the animal system as early and as certainly as possible under the influence of mercury, proposes the employment of what he calls a normal solution of mercury, (*liqueur mercurielle normale,*) and which is composed as follows:—

℞ Bichloride of mercury, four and half grains;
Chloride of sodium, fifteen grains;
Muriate of ammonia, fifteen grains;
The white of an egg;
Distilled water, a pint.

Beat the white of egg with the distilled water; filter; dissolve the three ingredients in the fluid, and filter anew.

In an ounce of the above solution there is consequently rather more than a quarter of a grain of bichloride of mercury.—*Lancet, from Journ. des Coun. Med. Prat.*

---

### STATISTICS OF FRACTURES.

It appears, from the report of MM. Manoury and Thore, that in 1841 there were treated in the Hôtel Dieu, 109 cases of fractures, of which 40 occurred in January, May, and October. Out of the entire number, also, 8 had fractures of more than one bone, viz., 5 had 2, 2 had 3, and 1 had 4 fractures,—seven of these subjects having received their injuries by falls from high places; the multiplicity of fractures, however, in the same subject not having been found to retard the cure. With respect to the proportion of members fractured, it appears that out of 121 cases, 36 were fractures of the superior members; 54 of the inferior; 12 of the skull; and 19 of the spine and trunk; and further, comparing the fractures according to the sides of the body in which they occurred, 64 were found on the left, and only 39 on the right side,—a very different statement from that of Dupuytren, who assigns 7-10ths of these fractures to the right, and only 2-10ths to the left side. The small number of cases, however, from which the former conclusion is drawn makes it obviously of little value. MM. Manoury and Thore further state that of the fractures of the limbs, (most of which are caused by direct violence,) 20 were of the right and 19 of the left arm; 35 of the left and 19 of the right leg. The fractures of the clavicle were seven; all on the left side. The eight fractures of the skull were all fatal.—*Ibid, from L'Experience.*

